# The Rochester Public Health Association

**Published:** 1912-04

**Authors:** E. G. Whipple

**Affiliations:** Executive Secretary


### The Rochester Public Health Association.

THE work of the Rochester Public Health Association is assuming such proportions, that the present quarters are entirely inadequate. The immediate cause of the work is due to the revival of the General Clinic, through which all children must go before they are assigned to a special clinic. This general clinic acts as a sort of clearing house, so that no abnormal condition, which has a tendency to lower the resistance of the child, is overlooked. Schedules of the clinics have been sent to all teachers and principals of the schools and to the mother's clubs, and through these sources a large number of children come to the Dispensary daily. A pamphlet is being prepared, giving to the teachers and mothers the symptoms of conditions which are treated at the Dispensary. Abnormalities of the eye, ear, nose, throat, nervous system, osseous system and early symptoms of tuberculosis are briefly touched upon in such language that it is readily understood.

Children predisposed to tuberculosis, those anemic and poorly nourished are being cared for by the Open Air School. This school is conducted by the Board of Education of the City of Rochester and the Rochester Public Health Association. The report for the last term, September to January, shows an average gain in weight of 4.925 pounds, and a total gain of 147¾ pounds. There was no child in the school who lost weight. Many children are constantly being returned to the public schools, where they take up the work of the same grade they would have been in, had they remained in the public school. The increase in percentage of haemoglobin had been gradual and apparent in almost every child, undoubtedly due to the regime of fresh air, rest and additional food.

The tuberculosis clinic shows a gradual increase in the number of visits to the Dispensary and also in the number of visits made by the tubcreulosis visiting nurses. The most striking feature of this clinic is the large percentage of family cases which are found to be tuberculous. This has varied from 10 per cent. to 40 per cent. of those examined. This represents examinations of persons in the families in which there exists a known case of tuberculosis.

There is close co-operation between the Rochester Public Health Association and Iola Sanitorium, the County Tuberculosis Hospital of Monroe County. Cases are discovered through the Health Association and sent to the Hospital for treatment. When discharged from the Hospital, they are placed under the supervision of the Health Association. Every admission into the Hospital is reported to the Health Association, which immediately sends out the tuberculosis visiting nurse to the house occupied by the patient. Here proper methods of disinfecting are carried out and an attempt is made to get other members of the family to come to the Dispensary. In return a report is made to the Hospital as to the condition of the home and other members of the family. The Hospital is already accommodating nearly 100 patients and has a capacity but for 60, and there now exists a large waiting list.

A schedule of clinics and a roster of the physicians of the Dispensary Staff shows the scope of the work.

E. G. WHIPPLE, M. D.,
*Executive Secretary.*

